# Blood pressure and low-density lipoprotein cholesterol control status in Chinese hypertensive dyslipidemia patients during lipid-lowering therapy

**DOI:** 10.1186/s12944-019-0974-y

**Published:** 2019-01-29

**Authors:** Xiaowei Yan, Yong Li, Yugang Dong, Yanhua Wu, Jihu Li, Rui Bian, Dayi Hu

**Affiliations:** 1 Department of Cardiology, Peking Union Medical College Hospital, No.1 Shuaifuyuan Wangfujing Dongcheng District, Beijing, 100730 China; 2 Department of Cardiology, Huashan Hospital, Shanghai Medical College, Fudan University, No. 12, Wulumuqi Rd. (middle), Shanghai, 200040 China; 3 Department of Cardiology, The First Affiliated Hospital, Sun Yat-sen University, No. 58, Second Zhongshan Road, Guangzhou, 510080 China; 4 Medical Affairs, Merck Sharp & Dohme (China) Holding Ltd. Building A, Headquarters Park Phase 2, 1582 Gumei Road, Xuhui District, Shanghai, 200233 China; 5 Outcome Research, Merck Sharp & Dohme (China) Holding Ltd. Building A, Headquarters Park Phase 2, 1582 Gumei Road, Xuhui District, Shanghai, 200233 China; 6 Department of Cardiology, Peking University People’s Hospital, No. 11 Xizhimen South Street Xicheng District, Beijing, 100044 China

**Keywords:** Blood pressure status, DYSIS, Dyslipidemia, Hypertension, Low-density lipoprotein cholesterol

## Abstract

**ᅟ:**

The present study comprised 17,096 Chinese hypertensive dyslipidemia patients who received lipid-lowering treatment for > 3 months in order to investigate blood pressure (BP) as well as low-density lipoprotein cholesterol (LDL-C) goal attainment rates in Chinese hypertensive dyslipidemia patients on antidyslipidemia drugs. The factors that interfered with BP, or BP and LDL-C goal attainment rates and antihypertensive treatment patterns, were analyzed. In total, 89.9% of the 17,096 hypertensive dyslipidemia patients received antihypertensive medications mainly consisting of a calcium channel blocker (CCB) (48.7%), an angiotensin receptor antagonist (ARB) (25.4%) and an angiotensin-converting enzyme inhibitor (ACEI) (15.1%). In cardiology departments, usage rates of β-blockers (19.2%) were unusually high compared to other departments (4.0–8.3%), whereas thiazide diuretics were prescribed at the lowest rate (0.3% vs 1.2–3.6%). The overall goal attainment rates for combined BP and LDL-C as well as BP or LDL-C targets were 22.9, 31.9 and 60.1%, respectively. The lowest BP, LDL-C and BP combined with LDL-C goal attainment rates were achieved in endocrine departments (19.9, 48.9 and 12.4%, respectively). Combination therapies showed no benefit particularly for BP goal achievement. A multivariate logistic regression analysis showed that age < 65 years, alcohol consumption, diabetes, coronary heart disease (CHD), cerebrovascular disease (CVD), chronic kidney disease (CKD), body mass index (BMI) ≥ 28 kg/m^2^ and not achieving total cholesterol goals were independent predictors for achieving BP, LDL-C or combined BP and LDL-C goals. In summary, the BP and LDL-C goal achievement rates in Chinese dyslipidemia outpatients with hypertension were low, especially in endocrine departments. Combination therapies were not associated with improvement of the goal achievement rates.

**Trial registration:**

Clinical trial registration number NCT01732952

**Electronic supplementary material:**

The online version of this article (10.1186/s12944-019-0974-y) contains supplementary material, which is available to authorized users.

## Introduction

The China Hypertension Survey of 2012–2015 revealed that 23.2% of adult Chinese people were hypertensive [1] and during 2013 and 2014 the prevalence of high total cholesterol, high low-density-lipid cholesterol, low high-density-lipid cholesterol and high triglycerides in China were 6.9, 8.1, 20.4 and 13.8%, respectively [2]. According to a recent Chinese study, the most detected comorbidities with hypertension were coronary heart disease (21.71%), diabetes (16.00%) and hyperlipidemia (13.81%) [3]. A rise of 20 mmHg in systolic blood pressure (BP) and 10 mmHg for diastolic BP from baseline has been shown to be associated with an increased risk of stroke, but only a rise of systolic BP from baseline increased the risk of myocardial infarction (MI) [4]. The cumulative risks of combined hypertension and dyslipidemia are considered to be higher than the summed risks from hypertension and dyslipidemia alone in cardiovascular disease (CVD) and coronary heart disease (CHD [5]. In a previous study, it was estimated that optimally controlled BP would prevent 37% of CHD events, optimally controlled blood cholesterol would prevent 62%, and a combination of both would lead to a 76% reduction of CHD events [6]. A recently study reported that a combination of cholesterol lowering agents with antihypertensive drugs led to a significantly lower rate of cardiovascular events compared to solely using cholesterol lowering therapy [7]. However, previous surveys revealed that the BP attainment rates of Chinese hypertension patients were as low as 18–20% [8, 9]. The DYSIS-China [10] databank from 2012 collected data about dyslipidemia patients from mainland China and enabled us to analyze the prevalence of hypertension, BP and low-density lipoprotein cholesterol (LDL-C) goal attainments in dyslipidemia patients with concomitant hypertension. In addition, we hypothesized that differences in hospital departments might have influenced the BP and LDL-C target attainment rates in dyslipidemia patients with hypertension.

## Patients and methods

DYSIS-China is one part of a DYSIS series of epidemiological studies, which is a cross-sectional observational study when the clinical data of enrolled patients are collected and recorded but the treatments and clinical evaluations are unaffected. The patients’ data were collected from departments of cardiology, endocrinology, geriatrics, internal medicine and neurology as well as other departments (including departments of nephrology, hematology, gastroenterology, immunology, respiratory diseases, infectious diseases, general internal medicine and traditional Chinese medicine) in Tier 1, Tier 2 and Tier 3 hospitals. For the DYSIS-China study, data from 25,311 dyslipidemia patients who were treated with lipid lowering drugs, including a large percentage of patients with hypertension, were collected from 122 hospitals in 6 geographic regions of China between April 2012 and October 2012. Patients were consecutively enrolled in the study if they were older than 45 years and had accepted lipid lowering drug therapy for ≥ three months. Demographic data, cardiovascular risk factors, a history of cardiovascular disease and corresponding treatments were recorded through a disease history inquiry, and laboratory test results were also collected. Laboratory measurements included the fasting lipid profile [TC, triglyceride (TG), LDL-C, high-density lipoprotein-cholesterol (HDL-C)], fasting blood-glucose, glycosylated-hemoglobin and serum creatinine. In addition, each patient’s hospital Tier and the treating department were noted.

For this study, 17,096 (67.5%) patients with concomitant hypertension from the DYSIS-China database were selected and we analyzed their antihypertensive treatments, LDL-C and BP goal attainments. Hypertension was defined as patients with a previous diagnosis of hypertension according to the criteria devised by Liu et al. (2011) [11], independent of antihypertensive therapy.

The BP and LDL-C target values were based on the 2010 Chinese hypertension management guidelines [11] and the Chinese 2007 dyslipidemia recommendations [12], with a target SBP/DBP < 140/90 mmHg for uncomplicated hypertension, < 150/90 mmHg for the elderly (≥ 65 years) and < 130/80 mmHg for those with diabetes, coronary heart disease or renal disease. LDL-C treatment goals were < 4.1 mmol/L (160 mg/dL), < 3.4 mmol/L (130 mg/dL), < 2.6 mmol/L (100 mg/dL) and < 2.0 mmol/L (80 mg/dL) for low-, moderate-, high- and very high-risk patients, respectively. TC treatment goals were < 6.2 mmol/L (240 mg/dL), < 5.2 mmol/L (200 mg/dL), < 4.1 mmol/L (160 mg/dL), and < 3.1 mmol/L (120 mg/dL) for low-, moderate-, high- and very high-risk patients, respectively. Monotherapy to lower BP included ACEI, ARA, thiazide diuretics, β-blockers or CCB. Combination therapy used at least two of the afore-mentioned drug classes to control BP.

Patients who were taking part in other clinical research trials were excluded from the DYSIS-China study. Each patient signed an informed consent form. The ethical committee of each research center involved in the DYSIS-China survey approved the research project and the contents of the informed consent form.

### Statistical analysis

Categorical variables are presented as frequencies and percentages, and continuous variables as the mean ± standard deviation (SD). Intergroup differences in categorical variables were evaluated using a chi-squared analysis or Fisher’s exact test, depending on the number of patients in each group. Intergroup differences in continuous variables were determined using analysis of variance. Multiple logistic regression analysis was performed to determine the independent predictors of lipid parameters. The variables included in the model were: age ≥ 65 years, gender, smoker, alcohol drinker, cardiovascular family history, BMI ≥ 28 kg/m^2^, diabetes mellitus (DM), coronary artery disease (CAD), cerebrovascular disease (stroke), ≥ stage 3 chronic kidney disease (CKD), TC not at goal, LDL-C not at goal, and combination therapy vs monotherapy; backward elimination (α = 0.05) was performed. Statistical significance was accepted at the two-sided 0.05 level and all confidence intervals were computed at the 95% level. Statistical analyses were performed using SAS (ver. 9.2, SAS Institute Inc., Cary, NC, US).

## Results

### Characteristics of study subjects

The DYSIS-China database was used to compare the characteristics of hypertensive and non-hypertensive subjects in the dyslipidemia population. The average course of hypertension was 10.7 ± 9.7 years in 17,096 dyslipidemia patients with hypertension, and comorbidities were diabetes in 6289 (36.8%), CHD in 6958 (40.7%) and BMIs ≥28 kg/m^2^ in 2658 (15.6%) cases. Hypertension treatment modalities were 50.5% monotherapy and 39.4% combination therapy. Statins were used as medication for 15, 363 (89.9%) and antiplatelet therapies for 11,989 (70.1%) hypertensive dyslipidemia patients (Table 1).

**Table 1:** Characteristics of dyslipidemia patients with hypertension in the DYSIS-China database

Index	With hypertension
	(*n* = 17,096)
Age (years)	
Mean (SD)	66.6 (10.4)
≥ 65 years	9769 (57.1%)
Gender	
Female	8226 (48.1%)
BMI [Mean (SD)] (kg/m^2^)	25.0 (3.3)
BMI ≥ 28 kg/m^2^	2658 (15.6%)
Course of hypertension [Mean (SD)] (year)	10.7 (9.7)
Smoking	
Current smoker	2105 (12.3%)
Previous smoker	3099 (18.1%)
Non-smoker	11,892 (69.6%)
Alcohol consumption	
Yes	1572 (9.2%)
No	13,795 (80.7%)
Gave up	1729 (10.1%)
Sedentary lifestyle	3595 (21.0%)
Family history of cardiovascular disease	1744 (10.2%)
Systolic pressure [Mean (SD)] (mmHg)	135.5 (15.8)
Diastolic pressure [Mean (SD)] (mmHg)	80.1 (10.2)
TC [Mean (SD)] (mmol/L)	4.5 (1.2)
LDL-C [Mean (SD)] (mmol/L)	2.6 (1.0)
HDL-C [Mean (SD)] (mmol/L)	1.3 (0.4)
TG [Mean (SD)] (mmol/L)	1.9 (1.4)
Non HDL-C [Mean (SD)] (mmol/L)	3.3 (1.1)
Uric acid [Mean (SD)] (μmol/L)	335.4 (99.7)
Creatinine [Mean (SD)] (μmol/L)	81.7 (35.8)
CKD	1502 (8.8%)
Diabetes	6289 (36.8%)
CHD	6958 (40.7%)
CVD	3363 (19.7%)
Heart failure	767 (4.5%)
Peripheral vascular disease	215 (1.3%)
Number of comorbidities^a^	
0	4597 (26.9%)
1	7522 (44.0%)
2	3659 (21.4%)
> 3	1318 (7.7%)
Antihypertension treatment	15,373 (89.9%)
Monotherapy	8641 (50.5%)
Combination therapy	6732 (39.4%)
Antiplatelet therapy	11,989 (70.1%)
Statin treatment	15,363 (89.9%)
Tier of Hospitals	
Tier 1	4117 (24.1%)
Tier 2	4433 (25.9%)
Tier 3	8546 (50.0%)
Department	
General medicine	4476 (26.2%)
Geriatric	2034 (11.9%)
Endocrine	2246 (13.1%)
Other	1199 (7.0%)
Neurology	2062 (12.1%)
Cardiology	5079 (29.7%)

^a^ The comorbidities included CKD, chronic kidney disease; CHD, chronic heart disease; CVD, cerebrovascular disease, diabetes and heart failure as well as peripheral vascular disease

SD, standard deviation; TC: total cholesterol; TG, triglyceride; CKD, chronic kidney disease; CHD, coronary heart disease; CVD, cerebrovascular disease; LDL-C, low-density lipoprotein cholesterol; HDL-C, high-density lipoprotein-cholesterol; BMI, body mass index. Other: departments except general medicine, geriatric, endocrinology, neurology and cardiology

### Medication treatment and goal attainment rates of BP and LDL-C in the hypertensive dyslipidemia population

**Fig. 1:**
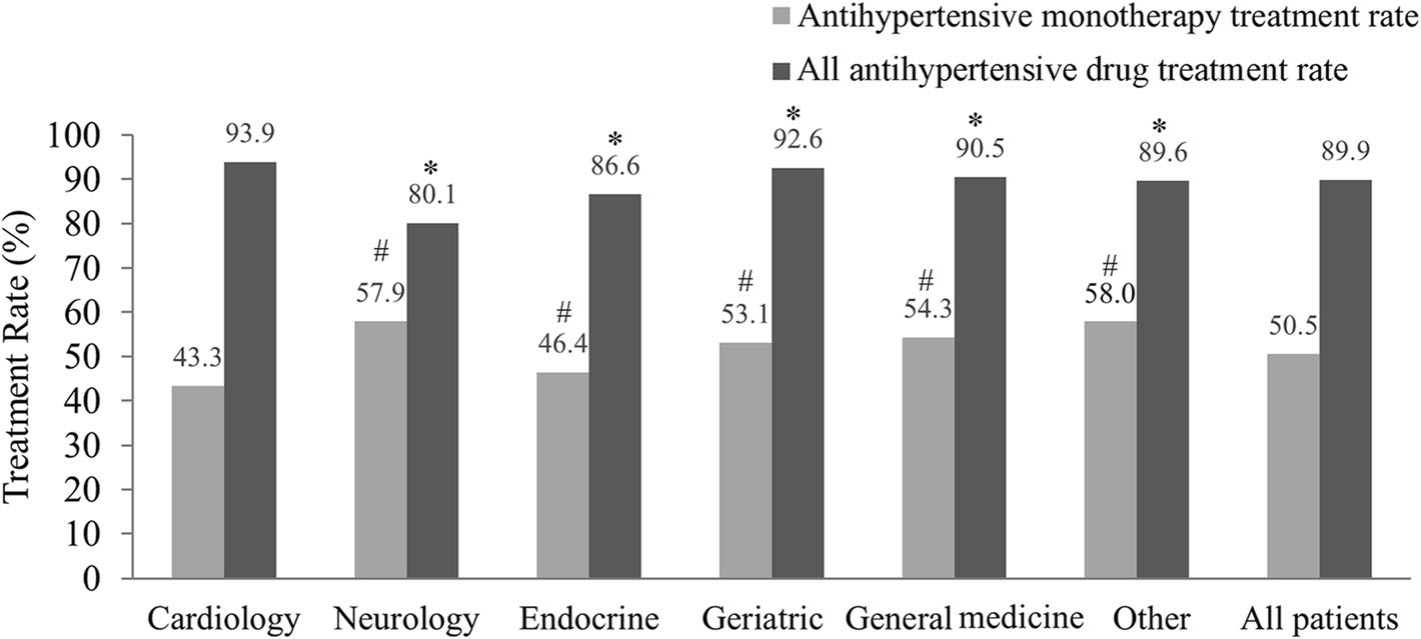
Distribution of patients prescribed antihypertensive treatment in different hospital departments. The monotherapy rate of the cardiology departments was significant different from other departments, #*P* < 0.01 compared with cardiology departments. The antihypertensive drug treatment rate was significantly different among the departments. It was higher in cardiology than in neurology, endocrinology, general medicine and other departments, *P < 0.01 compared with the cardiology department. Other: departments except general medicine, geriatric, endocrinology, neurology and cardiology.

**Fig. 2:**
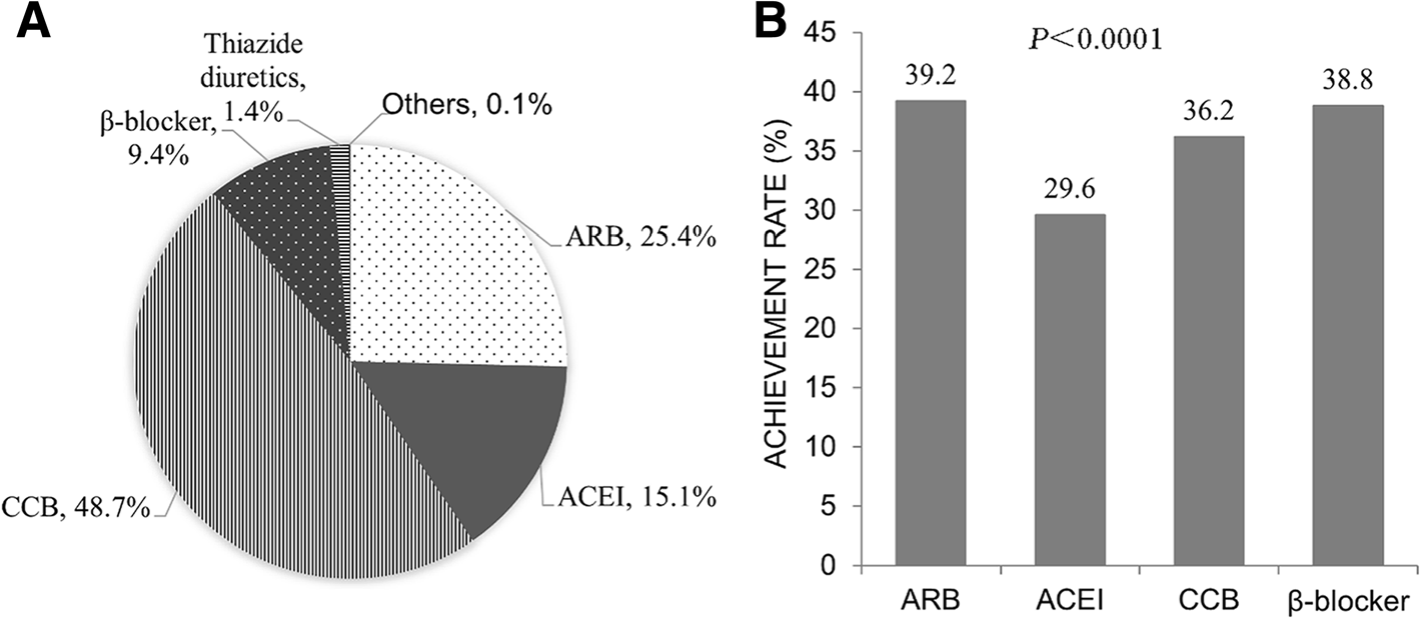
**a**, The percentage of antihypertensive drug distribution in patients with monotherapy; **b**, The blood pressure goal attainment rates for study patients prescribed various antihypertensive monotherapy. Since diuretics were used as monotherapy in only 1.4% of patients in this population, we excluded diuretics from Fig. 2. The blood pressure control rates among the various monotherapies were significantly different (*P* < 0.001)

First, we investigated the antihypertensive treatment rates in different hospital departments and found that the antihypertensive drug treatment rates were lowest in neurology (80.1%) and highest in cardiology (93.9%) departments (Fig. 1, Additional file 1: Table S1). Monotherapy was the main treatment mode (46.4–58.0%) in all departments apart from cardiology, in which 43.3% of patients received monotherapy and 50.6% combination therapy (Additional file 1: Table S1). The majority of combination treatments comprised 2 drugs (72.9–80.7%), followed by 3 drugs (17.2–23.1%), with very few patients treated with more than 3 drugs (1.1–4.1%) (Additional file 1: Table S1). The most frequently used medication for antihypertensive monotherapy was CCB (48.7%), followed by ARB (25.4%), ACEI (15.1%) and β-blockers (9.4%) (Fig. 2a), with the BP goal attainment rates being 36.2, 39.2, 29.6 and 38.8% for CCB, ARB, ACEI and β-blockers, respectively (Fig. 2b, Additional file 1: Table S1, *P* < 0.001). These data revealed that the BP achievements under monotherapy correlated with specific drugs, but the most efficient drugs were not the most frequently prescribed ones.

**Fig. 3:**
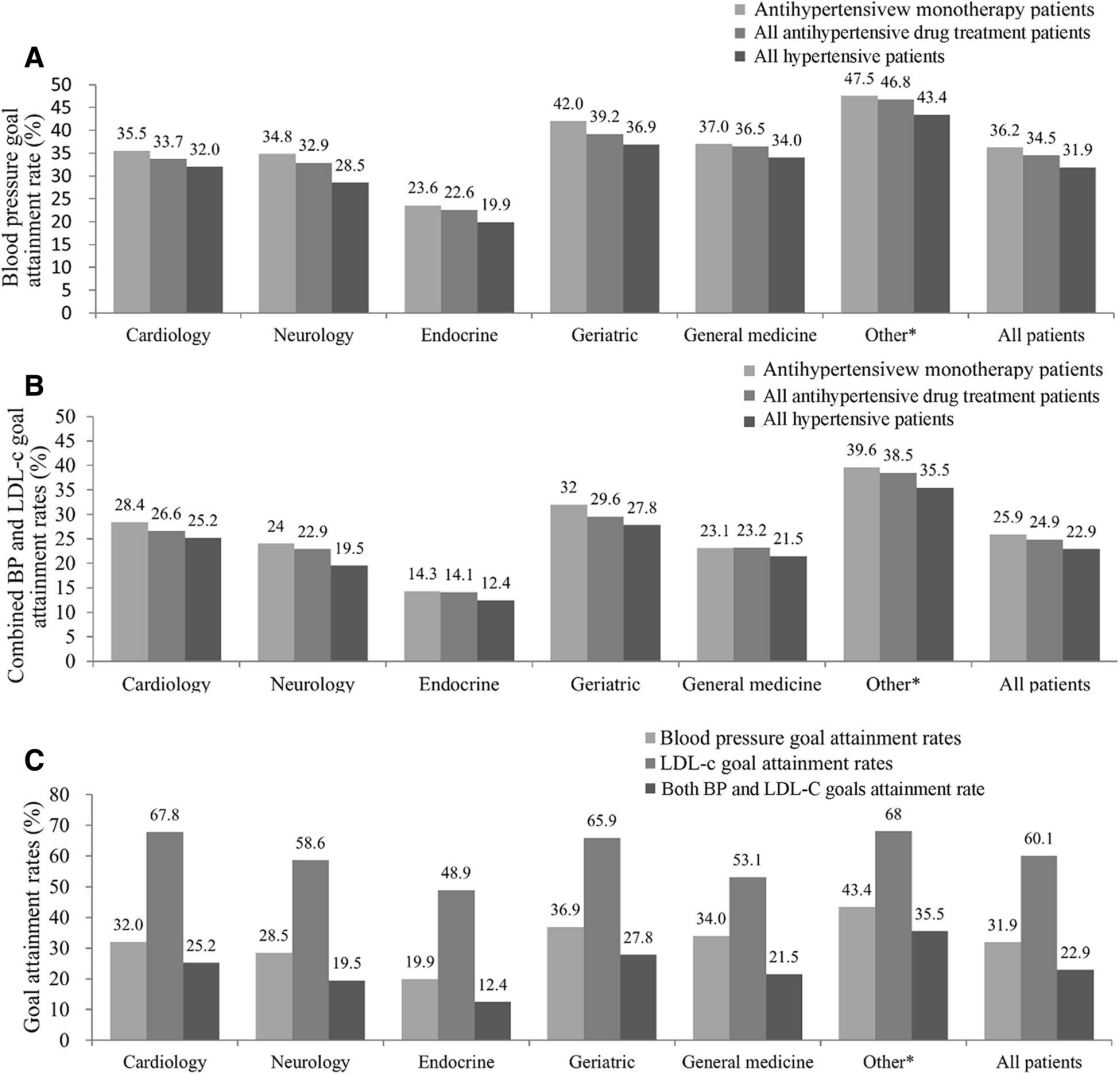
Blood pressure and LDL-C, and both BP and LDL-C goal attainment rates, of study patients in different departments. **a**: The blood pressure goal attainment rates of hypertension monotherapy patients, total antihypertensive treatment patients and all enrolled hypertensive dyslipidemia patients. **b**: Combined blood pressure and LDL-C goal attainment rates in hypertension monotherapy patients, all antihypertensive drug treatment patients and all enrolled hypertensive dyslipidemia patients. **c**: The blood pressure goal attainment rate, LDL-C goal attainments and BP combined with LDL-C goal attainment rate for all hypertensive dyslipidemia patients *P < 0.05, #*P* < 0.05, when compared with the department of cardiology. Other: departments except general medicine, geriatric, endocrinology, neurology and cardiology.

Then, we analyzed the distribution of drugs used in different clinical departments. ARB was most frequently used in endocrine departments, whereas ACEI (18.5%) and β-blockers (19.2%) were most frequently given in cardiology, and CCB was most widely used in neurology (65.1%), a value that is unusually high compared to other departments. All hypertensive dyslipidemia patients received lipid-lowering medication, with a statin being the most frequently prescribed drug (82.8–95.4%) in all departments (Additional file 1: Table S1). Next we analyzed the BP and combined BP and LDL-C goal attainment rates in monotherapy, all antihypertensive drug treatments and for all enrolled hypertensive dyslipidemia patients (Fig. 3a, b). We found that BP and LDL-C as well as combined BP and LDL-C goal attainment rates were the lowest in endocrine but highest in other departments (Fig. 3c).

### Univariate analysis of BP or LDL-C and combined goal achievements in dyslipidemia patients with hypertension

We investigated the BP and LDL-C goal attainment rates in the study population, based on their ages, different lifestyles, duration of hypertension, comorbidities and therapy in different hospitals and departments, or the type of hypertension treatment (Table 2). Patients ≥65 years old had higher BP and lower LDL-C goal attainment rates than those < 65 years of age. When the duration of hypertension was < 5 years, these patients had a higher BP or LDL-C and combined goal achievement rates comparable to those patients who had hypertension for ≥5 years. It was more difficult to control BP or LDL-C in current or previous smoking/alcohol consumption patients compared to those who did not smoke or drink. In patients with comorbidities, diabetics had the lowest BP (16.8%), LDL-C (46%) as well as combined BP and LDL-C (9.3%) goal attainment rates. For CKD, CHD, CVD, heart failure and peripheral vascular disease patients, combined BP and LDL-C goal attainments were all < 20%. Other factors leading to < 20% BP and LDL-C goal attainment rates were a BMI ≥ 28 kg/m^2^, a sedentary lifestyle and treatment in endocrine or neurology departments. The BP goal attainment rate in Tier 1 hospitals was the highest followed by Tier 2 and Tier 3 hospitals, while the LDL-C goal attainment rate in Tier 3 was the greatest followed by Tier 2 and Tier 1. The percentage of patients achieving BP goals was higher for individuals on antihypertensive monotherapy (36.2%) compared to those who received combination therapy (32.4%) (Table 2).

**Table 2:** Blood pressure and LDL-C goal attainment rates for 17,096 hypertension patients based on age, different life styles, duration of hypertension and treatment types in different Tier hospitals and departments

Index	Hypertension goal achievement % (N)	LDL-C goal achievement % (N)	Both BP and LDL-C goal achievement % (N)
Total (*n* = 17,096) Age (years)	31.9% (5450)	60.1% (10,281)	22.9% (3971)
≥ 65 (*n* = 9769)	34.6% (3377)	58.6% (5726)	23.6% (2302)
< 65 (*n* = 7327)	28.3% (2073)	62.2% (4555)	22.0% (1615)
*P*-value	< 0.001	< 0.001	0.019
Duration of Hypertension^a^ (years)			
< 5 (*n* = 5036)	34.8% (1754)	63.8% (3212)	26.0% (1310)
≥ 5 (*n* = 10,588)	32.9% (3485)	58.8% (6225)	23.2% (2460)
*P*-value	0.018	< 0.001	0.001
BMI ≥ 28 kg/m^2b^			
Yes (*n* = 2658)	25.1% (667)	55.8% (1482)	17.4% (462)
No (*n* = 14,432)	33.1% (4779)	60.9% (8794)	23.9% (3451)
*P*-value	< 0.001	< 0.001	< 0.001
Smoking			
Current smoker (*n* = 2105)	27.9% (587)	59.4% (1250)	20.0% (422)
Previous smoker (*n* = 3099)	28.3% (876)	63.6% (1972)	20.4% (631)
Non-smoker (*n* = 11,892)	33.5% (3987)	59.4% (7059)	24.1% (2864)
*P*-value	< 0.001	0.001	< 0.001
Drinking alcohol			
Yes (*n* = 1572)	24.6% (387)	59.2% (931)	19.0% (298)
No (*n* = 13,795)	33.4% (4602)	60.1% (8285)	23.8% (3288)
Gave up drinking (*n* = 1729)	26.7% (461)	61.6% (1065)	19.1% (331)
*P*-value	< 0.001	0.347	< 0.001
Sedentary lifestyle^b^			
Yes (*n* = 3595)	29.3% (1054)	58.0% (2086)	19.8% (712)
No (*n* = 13,500)	32.6% (4396)	60.7% (8195)	23.7% (3205)
*P*-value	0.000	0.004	< 0.001
Family history of cardiovascular disease^b^			
Yes (*n* = 1744)	32.7% (570)	60.8% (1060)	23.2% (404)
No (*n* = 15,348)	31.8% (4879)	60.1% (9218)	22.9% (3513)
*P*-value	0.448	0.561	0.795
Comorbidities			
CKD (*n* = 1502)	19.8% (297)	59.3% (890)	14.8% (222)
Diabetes (*n* = 6289)	16.8% (1059)	46.0% (2895)	9.3% (583)
CHD (*n* = 6958)	20.3% (1410)	54.9% (3821)	12.8% (889)
CVD (*n* = 3363)	28.4% (956)	53.3% (1793)	16.8% (566)
Heart failure (*n* = 757)	25.8% (198)	58.9% (452)	17.1% (131)
Peripheral vascular disease (*n* = 215)	22.3% (48)	55.3% (119)	13.5% (29)
*P*-value	< 0.001	< 0.001	< 0.001
Number of comorbidities			
0 (*n* = 4597)	58.8% (2703)	76.7% (3525)	48.3% (2219)
1 (*n* = 7522)	24.3% (1830)	57.0% (4289)	15.3% (1152)
2 (*n* = 3659)	18.3% (670)	50.8% (1860)	11.0% (403)
≥ 3 (*n* = 1318)	18.7% (247)	46.1% (607)	10.8% (143)
*P*-value	< 0.001	< 0.001	< 0.001
Tier of Hospitals			
Tier 1 (*n* = 4117)	36.0% (1483)	55.3% (2277)	24.6% (1011)
Tier 2 (*n* = 4433)	32.1% (1424)	60.3% (2672)	22.9% (1014)
Tier 3 (*n* = 8546)	29.8% (2543)	62.4% (5332)	22.1% (1892)
*P*-value	< 0.001	0.010	0.010
Department			
General medicine (*n* = 4476)	34.0% (1520)	53.1% (2375)	21.5% (963)
Geriatric (*n* = 2034)	36.9% (750)	65.9% (1341)	27.8% (566)
Endocrine (*n* = 2246)	19.9% (446)	48.9% (1098)	12.4% (278)
Neurology (*n* = 2062)	28.5% (588)	58.6% (1208)	19.5% (402)
Cardiology (*n* = 5079)	32.0% (1626	67.8% (3444)	25.2% (1282)
Other^c^(*n* = 1199)	43.4% (520)	68.0% (815)	35.5% (426)
*P*-value	< 0.001	< 0.001	< 0.001
Hypertension treatment			
Monotherapy (*n* = 8641)	36.2% (3125)	58.9% (5091)	25.9% (2241)
Combination therapy (*n* = 6732)	32.4% (2184)	63.4% (4268)	23.6% (1591)
*P*-value	< 0.001	< 0.001	< 0.001

Note: The Chi-squared test was used for categorical variables, while Fisher’s exact test was used only when the number of patients in some units was <5. BP achievement goals were SBP/DBP < 140/90 mmHg for uncomplicated hypertension, < 150/90 mmHg for the elderly (≥ 65 years) and < 130/80 mmHg for those with diabetes, coronary heart or renal disease. LDL-C achievement goals for LDL-C treatment were < 4.1 mmol/L for low risk, < 3.4 mmol/L for moderate risk, < 2.6 mmol/L for high risk and < 2.0 mmol/L for very high-risk patients. CKD, chronic kidney disease; CHD, chronic heart disease; CVD, cerebrovascular disease.^a^ indicates that data from 1472 patients were not available

^b^ Indicates that data for 6 patients were not available

CKD, chronic kidney disease; CHD, coronary heart disease; CVD, cerebrovascular disease; BP, blood pressure; LDL-C, low-density lipoprotein cholesterol; BMI, body mass index

^c^ Others were departments except general medicine, geriatric, endocrinology, neurology and cardiology

### Multivariate logistic regression analysis of the factors affecting the BP target goal attainment

Multivariate logistic regression analysis revealed that the main predictors for BP attainment failure were alcohol consumption, diabetes, CHD, CVD, CKD and a BMI ≥ 28 kg/m^2^, and not reaching TC and LDL-C goals (Table 3). A multivariate logistic regression analysis of the risk factors for not achieving both target goals of BP and LDL-C revealed that the majority of risk factors responsible for not achieving the BP target goal attainment also appeared in the combination achievement analysis (Table 3).

**Table 3:** Multivariate logistic regression analysis of the risk factors for blood pressure goal attainment failure

	BP target attainment		BP and LDL-C targets attainment	
Variate	OR (95% CI)	*P*-value	OR (95% CI)	*P*-value
Age ≥ 65 years	0.602 (0.542, 0.668)	< 0.001	0.695 (0.616, 0.785)	< 0.001
Alcohol consumption				
Yes	1.474 (1.216, 1.787)	< 0.001	1.372 (1.101, 1.711)	0.005
Family history of cardiovascular disease	0.788 (0.661, 0.938)	0.009	0.779 (0.634, 0.956)	0.017
Sedentary lifestyle	0.974 (0.860, 1.104)	0.683	0.991 (0.855, 1.148)	0.100
Tier of Hospitals				
Tier 1	0.918 (0.717, 1.176)	0.498	0.870 (0.649, 1.166)	0.351
Tier 2	1.041(0.893, 1.214)	0.608	1.030 (0.862, 1.231)	0.744
BMI ≥ 28 kg/m^2^	1.292(1.113, 1.499)	0.001	1.364 (1.141, 1.631)	0.001
Diabetes	3.407 (3.003, 3.865)	< 0.001	3.440 (2.931, 4.038)	< 0.001
CHD	3.450 (3.062, 3.888)	< 0.001	3.848 (3.323, 4.456)	< 0.001
CVD	1.200 (1.049, 1.373)	< 0.001	1.585 (1.342, 1.872)	< 0.001
CKD	2.504 (2.034, 3.083)	< 0.001	2.487 (1.939, 3.190)	< 0.001
TC not reached goal	1.367 (1.197, 1.562)	0.008	6.183 (5.442, 7.026)	< 0.001
LDL-C not reached goal	1.456 (1.270, 1.670)	< 0.001	–	

Note: age, non-alcohol consumption was used as the control for the alcohol consumption; BMI: BMI ≤ 28 kg/m^2^ was the control, TC and LDL-C: reaching goal attainment was used as the control; non-comorbidities served as the control for the diabetes CHD, CVD and CKD risk factors, Tier, sedentary lifestyle; family history of cardiovascular disease

OR, odds ratio; CI, confidence interval; CKD, chronic kidney disease; CHD, coronary heart disease; CVD, cerebrovascular disease; BP, blood pressure; LDL-C, low-density lipoprotein cholesterol; BMI, body mass index; TC: total cholesterol

## Discussion

Our study is the first in China to explore both BP and LDL-C attainment rates in dyslipidemia patients with concomitant hypertension. It is commonly accepted that dyslipidemia is associated with hypertension [13–17], findings in good agreement with the results obtained in the present investigation. The data revealed that about two thirds of dyslipidemia patients in the DYSIS-China database had hypertension and only one third of them had their BP controlled.

Ten percent of all patients with hypertension in our study did not receive any hypertension medication. Of those who received treatment, 50.5% were on monotherapy and 39.4% on combination therapy. The relatively high percentage of untreated and unsuccessful monotherapy cases indicates a clinical inertia and poor disease management, particularly regarding the switch to combination therapy for the treatment of hypertension [18]. In our study, endocrine departments achieved the lowest LDL-C (48.9%), BP (19.9%) and combined LDL-C and BP goal attainment rates (12.4%), which might be associated with the lowest hypertension treatment (86.6%) and lipid lowering treatment rates with statin (85.4%) in these departments.

Neurologists and endocrinologists mainly focus on the patient’s specialized condition and treatment (nervous system function, blood glucose control, etc.). The use of statins and the lack of attention to LDL-C or adopting small doses for fear of side effects may be the reasons for the low LDL-C achievement rate.

Similarly, a recent study showed that cardiologists were more likely to prescribe combination antihypertensive therapy than endocrinologists [19]. Thus fixed-dose combinations should be encouraged especially for diabetic, CHD and CKD patients, or those patients who have a heavy pill burden to improve treatment compliance and BP target rates. In one randomized, double-blind, multicenter comparison study it was reported that switching to a fixed-dose combination therapy of amlodipine/losartan 5 mg/100 mg was associated with significantly greater reductions in BP and superior achievement of BP goals compared with a maintenance dose of losartan 100 mg [20]. Although we have published the Chinese Hypertension Prevention and Treatment Guideline in 2010 and stressed the importance of antihypertensive treatment, many patients, even physicians, remain reluctant to take or cannot prescribe optimal combination therapy. Some patients even practice “Qigong”, “Tai Chi” or take traditional Chinese medicine instead of conventional antihypertensive drugs.

According to the hypertension guidelines, the majority of patients need combination therapy to reach their BP goal. Early and aggressive combination treatment, particularly for patients with comorbidities, is an effective method to help patients achieve their goals, as the combination of different classes of antihypertensive drugs with various mechanisms of action produce synergistic BP lowering effects [21–23]. In fact, the Chinese Hypertension Intervention Efficacy (CHIEF) trial included 13,542 hypertensive patients from 180 centers in China. The preliminary report of CHIEF revealed that combination treatments of a CCB with a diuretic, or a CCB plus an ARB for hypertensive patients, produced BP attainment rates of 72.1 and 72.6%, respectively [24]. In a community-based chronic disease management program held in Hebei province, the BP control rate rose from 8.9 to 77.2% in 41,000 hypertensive patients who followed standard antihypertensive procedure and who were strictly monitored for one year by 7000 general physicians [25].

In present study, a strange finding was that the monotherapy was associated with a higher BP control rate (36.2%) than combination therapies (32.4%) (*P* = 0.001), and the more the number of antihypertensive drugs, the lower the BP control rate. Possible explanations for this strange phenomenon could be poor treatment compliances with the increment of drug-tablets [26, 27], or an unconscionable combination of different classes of antihypertensive drugs which should cause the attention of physicians and patients in the clinical practice.

In patients unresponsive to BP lowering therapy, the poor compliance may also be due to costs, availability of professional guidance, and lack of family support [28, 29]. However, poor compliance has been reported to be a global problem for hypertension treatments and the authors proposed electronic monitoring, drug measurements as well as single-pill fixed-dose combinations and medication repacking as methods of improving adherence [30]. Now with the ongoing health care reform in China, a nationwide, highly effective chronic disease prevention and control system is being established. This is probably the only way to implement our guideline recommendations, to educate our physicians and patients effectively and to promote the control of hypertension and dyslipidemia substantially. Enhanced compliance may also be the reason for relatively better goal achievement rates in patients’ ≥ 65 years old in our study, a finding that has also been highlighted in previous studies [31, 32].

The extremely low rate of diuretics prescription (1.4%) may in part account for the low BP attainment rate in our study population, especially in patients receiving combination therapy. In China, it has been reported that 58.7% of hypertension patients were salt-sensitive, a percentage that may even be higher in diabetic and/or elderly patients. In these patients, it is difficult to control BP without a diuretic in their antihypertensive drug regimen, particularly in the north of China where salt intake is as high as 12–18 g/day [33]. Therefore, in order to improve the BP control rates in China for patients refractory to monotherapy, combination treatment containing a diuretic should be considered. And in patients whose BP remained uncontrolled after non-diuretic or a two-drug combination, further adjustment of the treatment regimen should include a diuretic.

Our multivariate logistic regression analysis showed that obesity, diabetes, coronary heart disease, cerebrovascular disease and chronic kidney disease were independent risk factors associated with BP target attainment failure. The combined goal attainment rate for both BP and LDL-C was very low (22.9%) in our hypertensive dyslipidemia patients. For those with diabetes, CHD and/or CKD, the lower BP target (< 130/80 mmHg) in the 2010 Chinese Hypertension Guideline may partially account for the disappointing BP attainment rates. However, the BP target rate in patients with obesity or cerebrovascular disease were also very low, though these patients shared the same BP target value (SBP/DBP < 140/90 mmHg) as uncomplicated hypertensives. Moreover, the analysis of dyslipidemia management in DYSIS-China also revealed that diabetes was a strong predictor of failure in attaining LDL-C and non-HDL-C goals [10]. Zhao’s result are in accordance with the findings of our multivariate logistic regression analysis, which showed that diabetes was an independent risk factor for not achieving BP and combined BP and LDL-C targets. Therefore, besides the stricter BP target value for these comorbidities, there must be other reasons (vide supra) that may account for the low BP target attainment rates. Further measures should be taken to spread the recommendations of our guidelines in order to improve BP and LDL-C control rate in patients with comorbidities. The doctors in endocrine or neurology departments should focus more on the control of BP and LDL-C in their patients, though the circumstances in other departments were also not optimal in our study.

Though in “Other Departments” the percentages of treated patients (55.9% for hypertension and 62.4% for lipid lowering drugs) was not the highest (Additional file 1: Table S1), the goal attainment rates for BP (43.4%) (Additional file 2: Table S2), LDL-C (68%) (Table 2) and both BP and LDL-C (35.5%) (Additional file 3: Table S3) were the highest among all the departments examined. A possible explanation might be that in “Other Departments” the prevalence of comorbidities and risk factors were lower and fewer patients needed to have their BP and LDL-C under 130/80 mmHg and 2.0 mmol/L, respectively.

The present study has several limitations. Since it was an observational cross-sectional investigation, long-term outcomes could not be assessed. In addition, the information of the patients’ compliance was not collected purposefully in DYSIS-China. Hence we could not analyze the patients’ adherence to medication precisely in the present study. Furthermore, all the patients enrolled in DYSIS-China had already received at least 3 months antidyslipidemia treatment (inclusion criteria for DYSIS-China) and the treatment rate of statins in this patient population was as high as 89.7%. If DYSIS-China would have enrolled dyslipidemia subjects consecutively and not eliminated patients without previous antidyslipidemia treatment, the statins’ treatment rate would have certainly been much lower than 89.7%, and the combined BP and LDL-C targets attainment rates even worse than those in the present study.

## Conclusions

Although the prevalence of hypertension in Chinese dyslipidemia patients was high, a considerable proportion of patients failed to achieve the BP target, as well as both BP and LDL-C targets. An incomplete management system, improper monotherapy, inappropriate diuretic prescription and poor treatment compliance may account for the unsatisfactory goal attainment rates in Chinese patients with both hypertension and dyslipidemia. The data from our study clearly suggest that the establishment of a sound management system for the treatment of hypertension and dyslipidemia should become an important healthcare strategy in China.

## Additional file

Additional file 1: **Table S1.** Distribution of study patients treated with antihypertensive and lipid lowering drug treatment in different departments. (DOC 45 kb)

Additional file 2: **Table S2.** Blood pressure goal attainment rates of study patients based on different antihypertensive or lipid-lowering treatments in different departments. (DOC 49 kb)

Additional file 3: **Table S3.** Both blood pressure and LDL-C goal attainment rates of study patients based on different antihypertensive or lipid-lowering treatments in different departments. (DOC 50 kb)
