# Supplementary material for: Blood pressure and low-density lipoprotein cholesterol control status in Chinese hypertensive dyslipidemia patients during lipid-lowering therapy
Source: Lipids Health Dis. 2019 Jan 29;18:32. doi: 10.1186/s12944-019-0974-y (PMC6352342; doi:10.1186/s12944-019-0974-y)
Supplement: Supplementary file 1 — Table S1. Distribution of study patients treated with antihypertensive and lipid lowering drug treatment in different departments. (DOC 45 kb) [file 12944_2019_974_MOESM1_ESM.doc]

Additional file 1: Table S1 Distribution of study patients treated with antihypertensive and lipid lowering drug treatment in different departments

|  | | Cardiology  *n* = 5,079 (%) | Neurology  *n* = 2,062 (%) | | Endocrine  *n* = 2,246 (%) | | | | Geriatric  *n* = 2,034 (%) | General medicine  *n* = 4,476 (%) | | Other#  *n* = 1,199 (%) | All patients  *n* = 17,096 (%) | *P*-value |
| --- | --- | --- | --- | --- | --- | --- | --- | --- | --- | --- | --- | --- | --- | --- |
| Antihypertensive drug treatment | 4,769 (93.9) | | 1,651 (80.1) | 1,946 (86.6) | | | | 1,884 (92.6) | | | 4,049 (90.5) | 1,074 (89.6) | 15,373 (89.9) | <0.001 |
| Monotherapy | 2,197 (43.3) | | 1,194 (57.9) | 1,043 (46.4) | | | 1,081 (53.1) | | | | 2,431 (54.3) | 695 (58.0) | 8,641 (50.5) | <0.001 |
| ARB | 555 (25.3) | | 247 (20.7) | 440 (42.2) | | | 321 (29.7) | | | | 478 (19.7) | 155 (22.3) | 2,196 (25.4) | <0.001 |
| ACEI | 407 (18.5) | | 103 (8.6) | 151 (14.5) | | | 165 (15.3) | | | | 408 (16.8) | 69 (9.9) | 1,303 (15.1) |  |
| CCB | 805 (36.6) | | 777 (65.1) | 370 (35.5) | | | 487 (45.1) | | | | 1,356 (55.8) | 409 (58.8) | 4,204 (48.7) |  |
| Thiazide diuretics | 7 (0.3) | | 17 (1.4) | 13 (1.2) | | | 16 (1.5) | | | | 41 (1.7) | 25 (3.6) | 119 (1.4) |  |
| β-blocker | 422 (19.2) | | 48 (4.0) | 68 (6.5) | | | 90 (8.3) | | | | 146 (6.0) | 37(5.3) | 811 (9.4) |  |
| Other* | 1 (0.0) | | 2 (0.2) | 1 (0.1) | | | 2 (0.2) | | | | 2 (0.1) | 0 (0.0) | 8 (0.1) |  |
| Combination therapy | 2,572 (50.6) | | 457 (22.2) | 903 (40.2) | | | 803 (39.5) | | | | 1,618 (36.1) | 379 (31.6) | 6,732 (39.4) | <0.001 |
| 2 drugs | 1,874 (72.9) | | 357 (78.1) | 683 (75.6) | | | 615 (76.6) | | | | 1,268 (78.4) | 306 (80.7) | 5,103 (75.8) | <0.001 |
| 3 drugs | 593 (23.1) | | 95 (20.8) | 190 (21.0) | | | 158 (19.7) | | | | 314 (19.4) | 65 (17.2) | 1,415 (21.0) |  |
| > 3 drugs | 105 (4.1) | | 5 (1.1) | 30 (3.3) | | | 30 (3.7) | | | | 36 (2.2) | 8 (2.1) | 214 (3.2) |  |
| Lipid-lowering treatment drugs | 5,079 (100.0) | | 2,062 (100.0) | 2,246 (100.0) | | 2,034 (100.0) | | | | 4,476 (100.0) | | 1,199 (100.0) | 17096 (100.0) | NA |
| Statin | 4,846 (95.4) | | 1,927 (93.5) | 1,918 (85.4) | | 1,896 (93.2) | | | | 3,783 (84.5) | | 993 (82.8) | 15,363 (89.9 | < 0.001 |

Note: The percentage was numerator divided by the denominator, denominator: number of cases in a group which used drugs; the numerator: number of target medication treatment. *P*-value: Comparing the difference of the specific drug-treatment pattern among the different departments using the chi-square test.

ARB, angiotensin receptor antagonist; ACEI, angiotensin-converting enzyme inhibitor; CCB, calcium channel blocker.

*Other medication: non-thiazide diuretics and β-blocker. # Other: departments except general medicine, geriatric, endocrinology, neurology and cardiology
