# Supplementary material for: Blood pressure and low-density lipoprotein cholesterol control status in Chinese hypertensive dyslipidemia patients during lipid-lowering therapy
Source: Lipids Health Dis. 2019 Jan 29;18:32. doi: 10.1186/s12944-019-0974-y (PMC6352342; doi:10.1186/s12944-019-0974-y)
Supplement: Supplementary file 2 — Table S2. Blood pressure goal attainment rates of study patients based on different antihypertensive or lipid-lowering treatments in different departments. (DOC 49 kb) [file 12944_2019_974_MOESM2_ESM.doc]

Additional file 2: Table S2. Blood pressure goal attainment rates of study patients based on different antihypertensive or lipid-lowering treatments in different departments

| **Blood pressure goal attainment rates** | | | | | | | | |
| --- | --- | --- | --- | --- | --- | --- | --- | --- |
|  | Cardiology  *n* (%) | Neurology  *n* (%) | Endocrine  *n* (%) | Geriatric  *n* (%) | General medicine  *n* (%) | Other#  *n* (%) | All patients  *n* (%) | *P*-value |
| Total dyslipidemia patients with hypertension | 1,626/5,079 (32.0) | 588/2,062 (28.5) | 446/2,246 (19.9) | 750/2,034 (36.9) | 1,520/4,476 (34.0) | 520/1,199 **(43.4)** | 5,450/17,096 (31.9) | < 0.001 |
| Received antihypertensive drug treatment patients | 1,606/4,769 (33.7) | 543/1,651 (32.9) | 440/1,946 (22.6) | 738/1,884 (39.2) | 1,479/4,049 (36.5) | 503/1,074 **(46.8)** | 5,309/15,373 (34.5) | < 0.001 |
| Monotherapy | 781/2,197 (35.5) | 415/1,194 (34.8) | 246/1,043 (23.6) | 454/1,081 (42.0) | 899/2,431 (37.0) | 330/695 **(47.5)** | 3,125/8,641 (36.2) | < 0.001 |
| ARB | 216/555 (38.9) | 95/247 (38.5) | 103/440 (23.4) | 136/321 (42.4) | 214/478 (44.8) | 96/155 **(61.9)** | 860/2,196 (39.2) | < 0.001 |
| ACEI | 127/407 (31.2) | 29/103 (28.2) | 27/151 (17.9) | 72/165 **(43.6)** | 106/408 (26.0) | 25/69 (36.2) | 386/1,303 (29.6) | < 0.001 |
| CCB | 271/805 (33.7) | 268/777 (34.5) | 92/370 (24.9) | 196/487 (40.2) | 507/1,356 (37.4) | 186/409 **(45.5)** | 1,520/4204 (36.2) | < 0.001 |
| Thiazide diuretics | 2/6 (33.3) | 6/15 (40.0) | 2/11 (18.2) | 6/12 **(50.0)** | 11/34 (32.4) | 10/24 (41.7) | 37/102 (36.3) | 0.673 |
| β-blocker | 165/422 (39.1) | 16/48 (33.3) | 21/68 (30.9) | 41/90 **(45.6)** | 60/146 (41.1) | 12/37 (32.4) | 315/811 (38.8) | 0.404 |
| Other medications* | 0/2 (0.0) | 1/4 (25.0) | 1/3 (33.3) | 3/6 (50.0) | 1/9 (11.1) | 1/1 **(100.0)** | 7/25 (28.0) | 0.294 |
| Combination therapy | 825/2,572 (32.1) | 128/457 (28.0) | 194/903 (21.5) | 284/803 (35.4) | 580/1,618 (35.8) | 173/379 **(45.6)** | 2,184/6,732 (32.4) | < 0.001 |
| 2 drugs | 628/1,874 (33.5) | 104/357 (29.1) | 151/683 (22.1) | 228/615 (37.1) | 438/1268 (34.5) | 140/306 **(45.8)** | 1,689/5,103 (33.1) | < 0.001 |
| 3 drugs | 169/593 (28.5) | 24/95 (25.3) | 38/190 (20.0) | 48/158 (30.4) | 128/314 **(40.8)** | 30/65 **(46.2)** | 437/1,415 (30.9) | < 0.001 |
| > 3 drugs | 28/105 (26.7) | 0/5 (0.0) | 5/30 (16.7) | 8/30 (26.7) | 14/36 **(38.9)** | 3/8 (37.5) | 58/214 (27.1) | 0.261 |
| lipid-lowering drugs treatment | 1626/5079 (32.0) | 588/2062 (28.5) | 446/2246 (19.9) | 750/2034 (36.9) | 1,520/4,476 (34.0) | 520/1199 (43.4) | 5,450/17,096 (31.9) | < 0.001 |
| Statin treatment | 1521/4862 (31.3) | 568/1946 (29.2) | 397/1964 (20.2) | 702/1912 (36.7) | 1,304/3,874 (33.7) | 431/1006 (42.8) | 4,923/15,564 (31.6) | < 0.001 |

Note: The percentage was numerator divided by the denominator, the denominator: number of cases in a group which used drugs; the numerator: number of goal attainment cases*.* *P*-value: Comparing the difference of BP goal attainment rates among the different departments using the chi-square test.

ARB, angiotensin receptor antagonist; ACEI, angiotensin-converting enzyme inhibitor; CCB, calcium channel blocker; BP, blood pressure; LDL-c, low-density lipoprotein cholesterol.

*Other medications: non-thiazide diuretics and α-adrenoceptor antagonists. # Other: departments except general medicine, geriatric, endocrinology, neurology and cardiology
