# Supplementary material for: Blood pressure and low-density lipoprotein cholesterol control status in Chinese hypertensive dyslipidemia patients during lipid-lowering therapy
Source: Lipids Health Dis. 2019 Jan 29;18:32. doi: 10.1186/s12944-019-0974-y (PMC6352342; doi:10.1186/s12944-019-0974-y)
Supplement: Supplementary file 3 — Table S3. Both blood pressure and LDL-C goal attainment rates of study patients based on different antihypertensive or lipid-lowering treatments in different departments. (DOC 50 kb) [file 12944_2019_974_MOESM3_ESM.doc]

Additional file 3: Table S3. Both blood pressure and LDL-c goal attainment rates of study patients based on different antihypertensive or lipid-lowering treatments in different departments

| **Bothe BP and LDL-C all achieved goal attainment rates** | | | | | | | | | |
| --- | --- | --- | --- | --- | --- | --- | --- | --- | --- |
|  | Cardiology  *n* (%) | Neurology  *n* (%) | Endocrine  *n* (%) | Geriatric  *n* (%) | General medicine  *n* (%) | Other#  *n* (%) | | All patients  *n* (%) | *P*-value |
| Total dyslipidemia patients with hypertension | 1,282/5,079 (25.2%) | 402/2,062 (19.5%) | 278/2,246 (12.4%) | 566/2,034 (27.8%) | 963/4,476 (21.5%) | 426/1,199 (35.5%) | | 3,917/17,096 (22.9%) | < 0.001 |
| Received antihypertensive drug treatment patients | 1267/4,769 (26.6%) | 378/1,651 (22.9%) | 275/1,946 (14.1%) | 558/1,884 (29.6%) | 941/4,049 (23.2%) | 413/1,074 (38.5%) | | 3,832/15,373 (24.9%) | < 0.001 |
| Monotherapy | 623/2,197 (28.4%) | 286/1,194 (24%) | 149/1,043 (14.3%) | 346/1,081 (32%) | 562/2,431 (23.1%) | 275/695 (39.6%) | | 2,241/8,641 (25.9%) | < 0.001 |
| ARB | 183/555 (33%) | 64/247 (25.9%) | 61/440 (13.9%) | 105/321 (32.7%) | 141/478 (29.5%) | 88/155 (56.8%) | | 642/2,196 (29.2%) | < 0.001 |
| ACEI | 95/407 (23.3%) | 21/103 (20.4%) | 19/151 (12.6%) | 54/165 (32.7%) | 73/408 (17.9%) | 22/69 (31.9%) | | 284/1303 (21.8%) | < 0.001 |
| CCB | 218/805 (27.1%) | 188/777 (24.2%) | 52/370 (14.1%) | 147/487 (30.2%) | 306/1,356 (22.6%) | 150/409 (36.7%) | | 1,061/4,204 (25.2%) | < 0.001 |
| Thiazide diuretics | 1/6 (16.7%) | 3/15 (20.0%) | 2/11 (18.2%) | 5/12 (41.7%) | 6/34 (17.6%) | 5/24 (20.8%) | | 22/102 (21.6%) | 0.644 |
| β-blocker | 126/422 (29.9%) | 10/48 (20.8%) | 15/68 (22.1%) | 33/90 (36.7%) | 36/146 (24.7%) | 9/37 (24.3%) | | 229/811 (28.2%) | 0.187 |
| Other* | 0/2 (0%) | 0/4 (0%) | 0/3 (0%) | 2/6 (33.3%) | 0/9 (0%) | 1/1 (100.0%) | | 3/25 (12.0%) | 0.030 |
| Combination therapy | 644/2,572 (25.0%) | 92/457 (20.1%) | 126/903 (14.0%) | 212/803 (26.4%) | 379/1,618 (23.4%) | 138/379 (36.4%) | | 1,591/6,732 (23.6%) | < 0.001 |
| 2 drugs | 492/1,874 (26.3%) | 72/357 (20.2%) | 101/683 (14.8%) | 172/615 (28%) | 286/1,268 (22.6%) | 114/306 (37.3%) | | 1,237/5,103 (24.2%) | < 0.001 |
| 3 drugs | 129/593 (21.8%) | 20/95 (21.1%) | 23/190 (12.1%) | 33/158 (20.9%) | 83/314 (26.4%) | 21/65 (32.3%) | | 309/1415 (21.8%) | 0.002 |
| > 3 drugs | 23/105 (21.9%) | 0/5 (0%) | 2/30 (6.7%) | 7/30 (23.3%) | 10/36 (27.8%) | 3/8 (37.5%) | | 45/214 (21%) | 0.186 |
| lipid-lowering drugs treatment | 1,282/5,079 (25.2%) | 402/2,062 (19.5%) | 278/2,246 (12.4%) | 566/2,034 (27.8%) | 963/4,476 (21.5%) | | 426/1,199 (35.5%) | 3,917/17,096 (22.9%) | < 0.001 |
| Statin treatment | 1,197/4,862 (24.6%) | 392/1,946 (20.1%) | 240/1,964 (12.2%) | 534/1,912 (27.9%) | 819/3,874 (21.1%) | | 349/1,006 (34.7%) | 3,531/15,564 (22.7%) | < 0.001 |

Note: The percentage was numerator divided by the denominator, the denominator: number of cases in a group which used drugs; the numerator: number of goal attainment cases. *P*-value: Comparing the difference of both BP and LDL-c goal attainment rates among the different departments using the chi-square test.

ARB, angiotensin receptor antagonist; ACEI, angiotensin-converting enzyme inhibitor; CCB, calcium channel blocker. BP, blood pressure; LDL-c, low-density lipoprotein cholesterol

*Other medications: non-thiazide diuretics and α-adrenoceptor antagonists. # Oher: departments except general medicine, geriatric, endocrinology, neurology, and cardiology.
